# Estimating the impact of patient-level risk factors and time-varying hospital unit on healthcare-associated *Clostridioides difficile* infection using cross-classified multilevel models

**DOI:** 10.1017/ice.2025.10356

**Published:** 2026-02

**Authors:** Jessica Lynn Webster, Claudine T. Jurkovitz, Brisa N. Sánchez, Stephen Eppes, Neal D. Goldstein

**Affiliations:** 1 Department of Epidemiology and Biostatistics, Drexel University, Dornsife School of Public Health, Philadelphia, PA, USA; 2 Institute for Research in Equity and Community Health, ChristianaCare Health Services Inc., Wilmington, DE, USA; 3 Department of Pediatrics, ChristianaCare, Wilmington, DE, USA; 4 Department of Microbiology & Immunology, College of Medicine, Drexel University, Philadelphia, PA, USA

## Abstract

**Objective:**

To deconstruct the multiple levels of risk factors for *Clostridioides difficile* infection, using multilevel models (MLMs) accounting for patient movement.

**Study Design and Setting:**

Case-control study of patients hospitalized in three acute care Delaware hospitals, December 2019–December 2023.

**Patients:**

Cases were patients aged ≥18 years who tested positive for hospital-onset *C. difficile* infection. Controls were patients aged ≥18 years hospitalized more than 72 hours, who did not test positive for *C. difficile* infection.

**Methods:**

Hierarchical and cross-classified MLMs were used to calculate odds of *C. difficile* infection based on patient-level risk factors and to evaluate the variation in odds of infection attributable to environmental risk factors using the hospital unit(s) a patient was assigned to during hospitalization.

**Results:**

Our study included 1,223 patients (249 cases, 974 controls). In both models, greater odds of infection were associated with antibiotic exposure [adjusted odds ratio (aOR) = 11.20, 95% confidence interval (CI) = 7.19, 17.40; aOR = 12.80, 95% CI = 8.46, 19.40 for hierarchical and cross-classified models respectively] and health insurance (aOR = 1.74, 95% CI = 1.12, 2.68; aOR = 1.62, 95% CI = 1.03, 2.53; public vs. private). Median odds ratios (MOR) for both models indicated greater relevance of between-unit heterogeneity in the outcome than health insurance but less than antibiotic exposure (MOR = 1.83, 95% CI = 1.56, 2.30 and 2.71 95% CI = 2.10, 4.06).

**Conclusion:**

Using multilevel methods accounting for patient movement, we found that while antibiotic use is the most important risk factor in patients that developed *C. difficile* infection, environmental risk factors are additionally important and should be considered in research involving hospitalized patients and healthcare-associated infections.

## Introduction

Infection with *Clostridioides difficile* is a leading cause of severe intestinal illness in hospitalized patients, accounting for an estimated 15% of all nosocomial infections.^1^ Much of the existing research on risk factors for *C. difficile* infection focus on antibiotic exposure, an inherently intrinsic, or patient-specific risk factor for symptomatic disease. Although this is often the most predictive factor for a patient experiencing disease from healthcare-associated *C. difficile* infection, it only captures one component of risk specific to the patient and their individual experiences. Environmental risk factors, specific to the larger environment or spatial unit a patient is existing within, may be equally as important in understanding transmission dynamics of healthcare-associated infections (HAIs),^2,3^ and a variety of prevention and control measures target this level of risk.^4–6^ These risk factors include occupancy or traffic of the healthcare facility, adherence to environmental cleaning practices, or frequency of antibiotic prescriptions. In a hospital, patients can be considered nested or clustered within rooms or units of the facility depending on where they have been placed throughout their stay. This spatial clustering could result in patients within the same physical space experiencing similar exposures and outcomes depending on these extrinsic pathways, which may add to or influence their patient-level risk factors such as age, medications, procedures or other comorbid conditions. For example, research has demonstrated increased risk of *C. difficile* infection in certain hospital units due to stewardship and infection prevention practices (increased antimicrobial prescribing) and patient risk factors (patients in intensive or long-term care units may be older, more likely to be prescribed antibiotics or other medications or have comorbidities).^7–9^ Adding to the complexity of working with hospital data and the reality of the patient’s experience during hospitalization is that patients will likely be exposed to different levels of environmental risk factors throughout their stay, due to movement (transfers) within and between clinical spaces. Thus, incorporating a time component in addition to a spatial component into such an analysis is important for correct identification of risk factors.

There are limited existing applications that consider clustering of patients within a healthcare facility,^10–12^ and even fewer in research involving HAIs.^13–16^ Through this work, we have identified a need for a practical application of multilevel modeling within the field of HAI research, highlighting methods that account for patient movement between and within healthcare facilities. There are several commonly used analytic options for evaluating risk factors that exist at different levels of data. Using a case-control study of hospitalized patients, we explore hierarchical and non-hierarchical (cross-classified) multilevel models (MLMs) to evaluate patient-level risk factors and the importance of environment on *C. difficile* infection, using time-varying hospital unit as a proxy for the environmental risk factors to account for patient movement and changing environments. We expect to see strong associations between patient-level risk factors, specifically antibiotic exposure, and *C. difficile* infection, but we additionally hypothesize that there will be variability in the odds of infection in patients between different units, suggesting environmental factors also play a role in a patient’s odds of becoming infected and developing disease. Through this work, we hope to provide clarity on how the distribution of HAIs such as *C. difficile* is affected by multiple levels of risk, with the goal of informing infection prevention measures on the unit-level and hospital-wide. This work will additionally provide methodological guidance to investigators conducting research on HAIs in a patient population moving within or between healthcare facilities throughout hospitalization.

## Methods

### Study population

Our study population included adults 18 years or older hospitalized at three ChristianaCare Health System facilities—coded Hospital A, Hospital B, and Hospital C—between December 2019 and December 2023. Hospitalized patients were directly admitted, transferred from another facility (including other acute care hospitals, skilled nursing, rehabilitation, assisted living, psychiatric assistance facilities, or the corrections department), or admitted via an emergency department. Data extracted from electronic health records (EHRs) included patient demographics, comorbidities, and time and date-stamped information on *C. difficile* infection, medications, procedures, and locations assigned throughout the facilities. Cases were patients admitted during the study period who tested positive for *C. difficile* infection by a two-step stool testing procedure [polymerase chain reaction (PCR) for *C. difficile* toxin B gene and enzyme immunoassay for toxins A and B] at least 72 hours after admission to ensure hospital- and not community-onset infection. Controls were any patient admitted during the study period with a length of stay (LOS) of at least 72 hours, who did not test positive for *C. difficile* infection (either did not test at all or tested negative). Time-stamped data for cases were collected from hospital admission until *C. difficile* infection, and for controls from hospital admission until discharge, transfer out of hospital system or death. Controls were randomly sampled from the hospital census using a 4:1 control to case ratio to maximize sample size. This study was reviewed and determined to be exempt from Institutional Review Board oversight by the ChristianaCare Institutional Review Board (IRB00000480).

### Covariates

We collected information from the EHR on demographic, health, and hospital stay characteristics and categorized them as either time-invariant patient-level or time-varying patient-level. Time-invariant patient-level variables included age at admission, sex, race/ethnicity, insurance, Agency for Healthcare Research and Quality (AHRQ) Elixhauser Comorbidity Measure^17^ and constructed variables summarizing the patient’s treatments during hospitalization: exposure to any antibiotics, number of unique antibiotic treatments, exposure to non-antibiotic medications (any chemotherapy, immunosuppressants, narcotic analgesic, steroid), number of unique non-antibiotic medications, any procedures, and overall LOS (days). Time-varying patient-level variables included timing of antibiotic treatment, non-antibiotic medications, procedures, and cumulative LOS (incorporated into daily calculation of probability of infection).

Additionally, several covariates were created based on the unit(s) visited by the patient. Time-invariant patient-level covariates included admission unit, and variables summarizing whether the patient visited a given unit, and a categorical variable for longest unit visit. The time-varying patient-level covariate included daily unit location. Units were categorized based on location and department. Some patients were assigned to more than four units during hospitalization, and while the specific unit(s) after the first four visited were not recorded within the data set, we specified when a patient had been assigned to five or more.

### Statistical analysis

Univariate and bivariate distributions of demographic, health, and hospital stay characteristics were assessed overall and by case-control status.

To determine the most appropriate analytic approach considering our research question, study population, and data availability, we evaluated the types of methods used for data with a multilevel structure. Table 1 and Supplemental Document 1 provide details on our process for selecting a multilevel modeling approach, along with a description of the methods assessed, data requirements, advantages and disadvantages of each method in the context of our research question, and a power calculation.

**Table 1.** Review of statistical methods that account for spatial- and time-unit clustering, model type, data structure, advantages, and disadvantages

Cluster	Model	Levels	Covariates	Advantages & disadvantages
Spatial-unit	Traditional GLM	Only patient	Patient-level covariates, unit-level covariates (including a unit identifier and/or variables specific to unit).	+ Ability to calculate the association between patient-level and unit-level covariates and the outcome, interactions between patient-level, unit-level covariates.
				**–** Outcome risk may be correlated between patients within units, standard errors may be incorrect due to lack of independence. Unable to account for time-varying unit-level covariates.
	Hierarchical MLM	L1: patient L2: unit	Patient-level covariates, unit-level covariates.	+ Ability to calculate the association between patient-level and unit-level covariates and the outcome, interactions between patient-level, unit-level covariates. Examine variability in odds of experiencing the outcome within units, between units.
				**–** Model complexity: requires greater number of observations to ensure model can be estimated. Unable to account for time-varying unit-level covariates due to hierarchical nature.
Time-unit	Hierarchical MLM	L1: patient-day L2: patient	Patient-day-level (time varying) covariates, patient-level covariates duplicated across rows, unit-level covariates either at the patient-day-level (time-varying) or patient-level (time-invariant).	+ Ability to calculate the association between patient-day-level covariates and the outcome, interactions between time-varying, patient-level covariates. Examine variability in odds of experiencing the outcome within patients, between patients.
				**–** Model complexity: requires greater number of observations to maintain power. Model assumes risk in one ward is independent of other wards, but this may be violated because patients travel from between units, standard errors may be incorrect due to lack of independence.
Both spatial- and time-unit	Hierarchical MLM	L1: patient-day L2: patient L3: unit	Patient-day-level (time-varying) covariates, patient-level covariates, unit-level covariates (time-invariant).	+ Ability to calculate the association between patient-day-level covariates and the outcome, interactions between time-varying, patient-level, and unit-level covariates. Examine variability in odds of experiencing the outcome within patients, between patients as well as within units, between units.
				**–** Model complexity: requires greater number of observations. Unable to account for time-varying unit-level covariates due to hierarchical nature.
	Cross-classified MLM*	L1: patient-day L2a: patient L2b: any unit	Patient-day-level (time-varying) covariates, patient-level covariates, unit-level covariates (time-invariant or time-varying).	+ Ability to calculate the association between patient-day-level covariates and the outcome, interactions between time-varying, patient-level, and unit-level covariates. Examine variability in odds of experiencing the outcome within patients, between patients as well as within units, between units. Able to account for time-varying unit-level covariates. Allows for the analysis of time-varying cluster units.
				**–** Model complexity: requires greater number of observations.

Note. GLM, generalized linear model, MLM, multilevel model, L1-2, level/group/cluster 1–2

**Figure 1.**
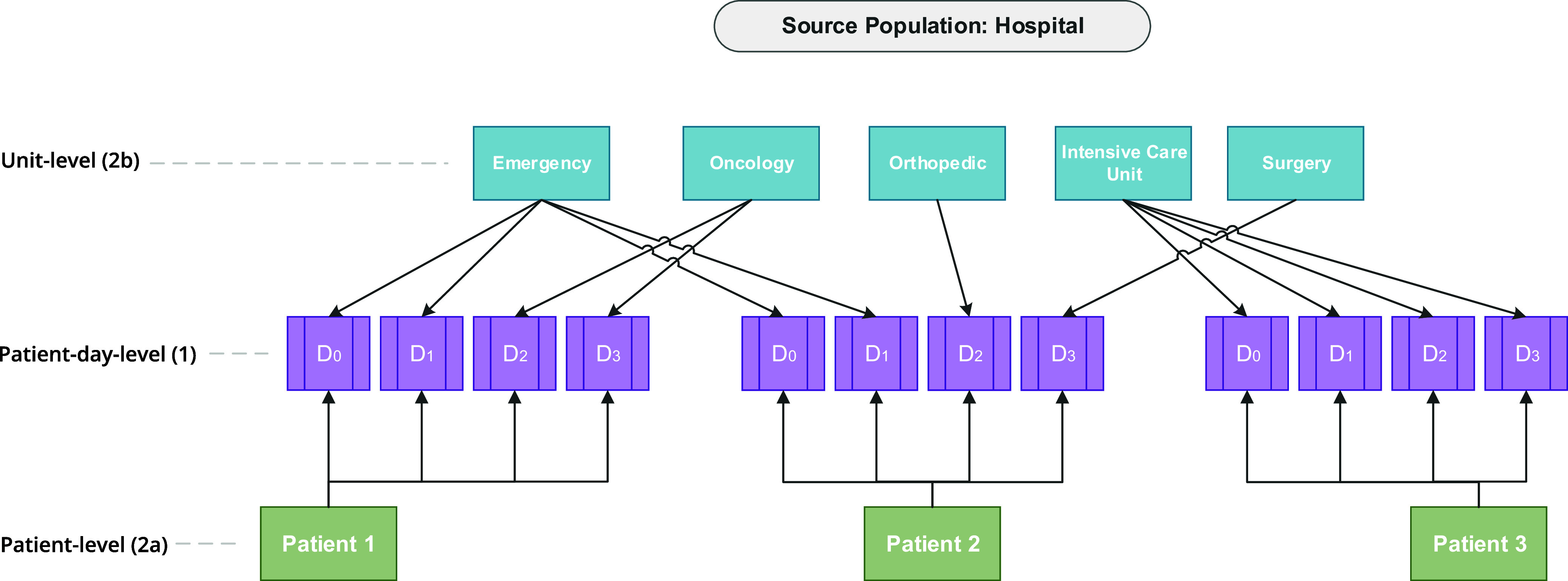
Diagram representing a cross-classified multilevel structure of hospitalized patients nested within time-varying hospital units using hypothetical data, incorporating patient movement by using patient day as the smallest unit of measurement.

We conducted two multilevel analyses: Model 1 was a two-level hierarchical MLM, with patients (level 1) nested within the unit where the patient spent the longest amount of time (level 2). This model accounts for correlation between patients who spent the longest amount of time in the same units, but ignores any potential correlation between patients within units where they spent less time. This method is commonly utilized in multilevel research accounting for a singular cluster unit per observation.^16^ Model 2 is a two-level cross-classified MLM, with level 1 representing patient-day, and two level 2 cluster units representing patient (level 2a) and unit (level 2b). A diagram representing this cross-classified multilevel structure is provided in Figure 1. This model allowed us to determine how much unit-level differences in *C. difficile* infection could be explained by patient-level risk factors. Equations used for each unadjusted and adjusted MLMs are provided in Supplemental Document 1.

We calculated adjusted odds ratios (aOR) and 95% confidence intervals (CI) of *C. difficile* infection, conditional on the patient and the unit. To quantify the variation in odds of *C. difficile* infection between units, we calculated the median odds ratio (MOR) for both models. The MOR is a method that transforms unit-level variance into the odds scale and can be used when the outcome of a MLM is binary. It is calculated by comparing the median odds of the outcome from two randomly chosen cluster units (i.e., two randomly chosen hospital units) and is interpreted as the extent to which the odds of infection differ based on which unit a patient is located in. This remaining difference in the odds of infection that exists after adjusting for covariates is referred to as residual heterogeneity. A MOR of 1 would indicate no variability in the odds of *C. difficile* infection between units, and the greater the MOR the greater the variability in odds of *C. difficile* infection between units.^18^ MOR CIs were calculated using 1,000 bootstrap replicates.

### Sensitivity analyses

Two sensitivity analyses were conducted: the first including a “season” cluster to account for potential seasonal trends in *C. difficile* infection, and the second using 1- and 2-day lagged time-varying covariate and exposure variables to account for the incubation period between infection and onset of symptoms or positive test.^13,19,20^

All analyses were conducted in RStudio (R version 4.2.2), MLM were run using the lme4 package.^21^ R code is available at https://github.com/jlywebster/cdiff_ccmlm.

## Results

From December 2019 to December 2023, there were 249 confirmed cases of hospital-onset *C. difficile* infection reported within the ChristianaCare hospital network. Our analytic sample included 1,223 patients (249 cases and 974 controls). Table 2 presents patient-level demographic, health, and hospital-stay characteristics overall and by case-control status, with time-varying variables summarized and aggregated to the patient-level. The patient’s unit cluster is represented as 1) any visit to each unit and 2) the unit where a patient spent the longest amount of time.

**Table 2.** Distribution of patient-level characteristics overall and by case-control status in patients hospitalized at ChristianaCare Hospitals in Delaware, 2019–2023

Variable	Overall		Cases		Controls	
	1,223	100%	249	20%	974	80%
**Demographic characteristics**						
Age at admission (Mean, SD)	63.2	(18.3)	66.7	(15.7)	62.2	(18.8)
Sex (female)	655	54%	131	53%	524	54%
Race/ethnicity						
NH White	781	64%	161	65%	620	64%
NH Black	343	28%	68	27%	275	28%
NH Asian or Pacific Islander	19	2%	6	2%	13	1%
NH American Indian or Alaska Native	3	0%	–	–	3	0%
Hispanic/Latino	65	5%	13	5%	52	5%
Other/unknown	12	1%	1	0.4%	11	1%
Insurance						
Private	310	25%	43	17%	267	27%
Public	913	75%	206	83%	707	73%
**Health characteristics**						
Number of AHRQ comorbidities (Median, IQR)	5	(7)	6	(7)	5	(7)
Any medications^a^ (yes)	802	66%	177	71%	625	64%
Number of unique medications^a^						
0	349	36%	72	29%	421	34%
1	217	22%	54	22%	271	22%
2	178	18%	53	21%	231	19%
3	156	16%	44	18%	200	16%
4+	74	8%	26	10%	100	8%
Any antibiotic exposure (yes)	623	51%	223	90%	400	41%
Number of unique antibiotic courses						
0	600	49%	26	10%	574	59%
1	331	27%	80	32%	251	26%
2	197	16%	86	35%	111	11%
3	80	7%	45	18%	35	4%
4	15	1%	12	5%	3	0%
Any medical procedures (yes)	846	69%	196	79%	650	67%
**Hospital stay characteristics**						
Length of stay, days (Median, IQR)	8	(7)	9	(10)	7	(6)
Admission unit						
Emergency room	966	79%	188	76%	778	80%
Physician referral	151	12%	29	12%	122	13%
Hospital transfer	70	6%	18	6%	52	5%
Unknown/not specified	36	3%	14	6%	22	2%
Number of units						
1	55	4%	18	7%	37	4%
2	489	40%	79	32%	410	42%
3	302	25%	55	22%	247	25%
4	191	16%	42	17%	149	15%
5+	186	15%	55	22%	131	13%

Note. IQR, interquartile range; SD, standard deviation; NH, non-Hispanic; AHRQ, Agency for Healthcare Research and Quality Elixhauser Comorbidity Measure.

a Chemotherapy, immunosuppressants, narcotic analgesics, and steroids (does not include antibiotics).

b Percentages will not add up to 100% as many patients visited multiple units.

Results from the MLMs are presented in Table 3. Model 1, the hierarchical MLM, resulted in higher odds of *C. difficile* infection when comparing patients who received antibiotics to those who did not (aOR = 11.20, 95% CI = 7.19, 17.40), patients with public health insurance to those with private (aOR = 1.74, 95% CI = 1.12, 2.68), and as LOS increased (aOR = 1.14, 95% CI = 1.00, 1.31). In Model 2, the cross-classified MLM, odds of *C. difficile* infection were higher when comparing patients who received antibiotics to those who did not (aOR = 12.80, 95% CI = 8.46, 19.40) and patients with public health insurance to those with private (aOR = 1.62, 95% CI = 1.03, 2.53).

**Table 3.**
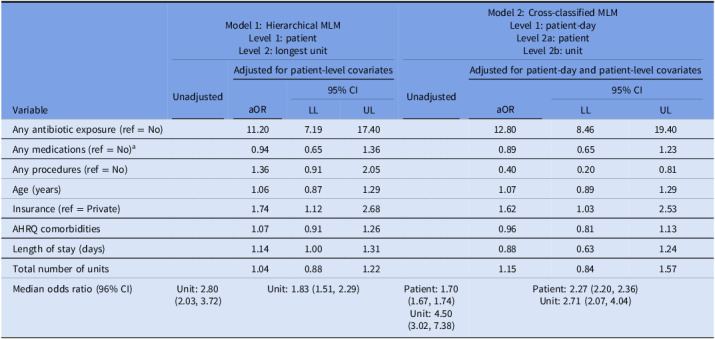
Unadjusted and adjusted hierarchical and nonhierarchical (cross-classified) multilevel generalized linear models of odds of *Clostridioides difficile* infection in a case-control study of patients hospitalized at ChristianaCare Hospitals in Delaware, 2019–2023

Note. MLM, multilevel model; aOR, adjusted odds ratio; CI, confidence interval; LL, lower limit; UL, upper limit; AHRQ, Agency for Healthcare Research and Quality Elixhauser Comorbidity Measure.

a Chemotherapy, immunosuppressants, narcotic analgesics, and steroids (does not include antibiotics).

In the adjusted Model 1, the MOR was 1.83 (95% CI = 1.51, 2.29), meaning that in the median case, the residual heterogeneity between units was 1.83 times that of the individual odds of *C. difficile* infection, when randomly comparing two patients in two different units. Comparing the MOR to the aORs, even after adjusting for patient-level factors, the residual heterogeneity between units (MOR = 1.83) was of greater relevance for understanding variations in the odds of *C. difficile* infection than the impact of a patient’s insurance (aOR = 1.74) and their LOS (aOR = 1.14), but of less relevance than antibiotic use (aOR = 11.20). In the adjusted Model 2, the MOR for patient was 2.27 (95% CI = 2.20, 2.36) and the MOR for unit was 2.71 (95% CI = 2.07, 4.04). This indicates that the residual heterogeneity between units (MOR = 2.71) was of slightly greater relevance for understanding variations in the odds of infection than that between patients (MOR = 2.27), as well as the impact of insurance (aOR = 1.62), but not as great as antibiotic use (aOR = 12.80).

### Sensitivity analyses

Results of the sensitivity analysis considering seasonal trends in infection are described in Table 4, and the sensitivity analysis considering lagged time-varying covariates in Table 5. Adjusted ORs for the patient-level associations changed marginally when accounting for season at admission or daily season cluster. Results of the second sensitivity analysis resulted in a more drastic change, with the odds of *C. difficile* infection based on antibiotic exposure 2.58 when lagged by 1 day and 2.23 when lagged by 2 days (compared to 12.80 without a lag).

**Table 4.**
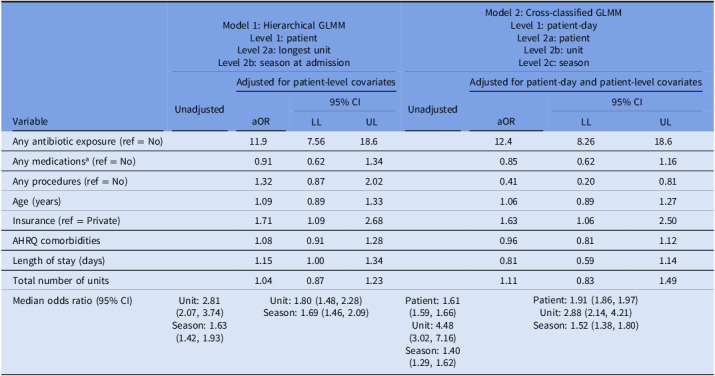
Sensitivity analysis to include season within unadjusted and adjusted hierarchical and nonhierarchical (cross-classified) multilevel generalized linear models of odds of *Clostridioides difficile* infection in a case-control study of patients hospitalized at ChristianaCare in Delaware, 2019–2023

Note. MLM, multilevel model; aOR, adjusted odds ratio; CI, confidence interval; LL, lower limit; UL, upper limit; AHRQ, Agency for Healthcare Research and Quality Elixhauser Comorbidity Measure.

a Chemotherapy, immunosuppressants, narcotic analgesics, and steroids (does not include antibiotics).

**Table 5.**
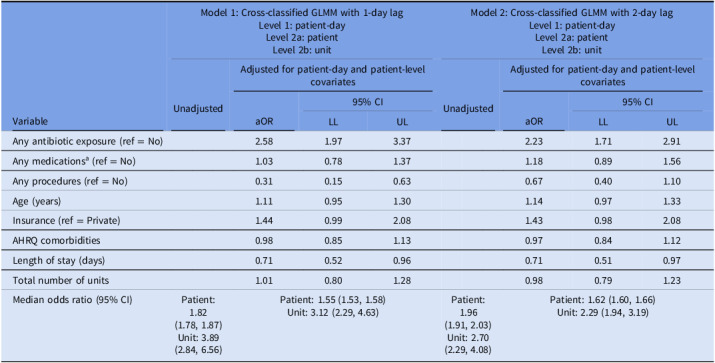
Sensitivity analysis using a 1- and 2-day lag on time-varying covariates within unadjusted and adjusted hierarchical and nonhierarchical (cross-classified) multilevel generalized linear models of odds of *Clostridioides difficile* infection in a case-control study of patients hospitalized at ChristianaCare Hospitals in Delaware, 2019–2023

Note. Lagged variables included any antibiotic exposure, any medications, any procedures, length of stay, and unit cluster.

MLM, multilevel model; aOR, adjusted odds ratio; CI, confidence interval; LL, lower limit; UL, upper limit; AHRQ, Agency for Healthcare Research and Quality Elixhauser Comorbidity Measure.

a Chemotherapy, immunosuppressants, narcotic analgesics, and steroids (does not include antibiotics).

## Discussion

This research sought to explore how multiple levels of risk factors may be associated with healthcare-associated *C. difficile* infection in a case-control study of hospitalized patients. Using a time-invariant hierarchical MLM and a time-varying cross-classified MLM, our results for the patient-level risk factors were consistent with existing literature—antibiotic exposure produced the greatest measure of association, followed by health insurance and LOS. However, LOS was only statistically significant in the time-invariant hierarchical MLM, meaning that once we accounted for patient movement throughout the hospital this factor was no longer associated with infection. This may be due to the way in which LOS was operationalized for the different models. For the hierarchical MLM, LOS represented the entire hospitalization, whereas in the cross-classified MLM, it was calculated as the cumulative number of days a patient had been hospitalized up to the given day of follow-up, perhaps providing a more accurate representation of timing of exposures in relation to the outcome. The importance of a patient’s location to their odds of infection was demonstrated when evaluating the residual heterogeneity between units. In both models, residual heterogeneity between units was less relevant than antibiotic exposure, but more relevant than health insurance and LOS for understanding the variation in patients’ odds of infection.

This study explores methods for working with multilevel clinical data extracted from EHR and identifies challenges that may arise and how to address them. The cross-classified MLM used in this analysis is common in studies that include individuals nested within fluid geographic spaces, such as neighborhoods or regions. However, we found that applications to clinical data, specifically clustering *within* hospital units were lacking, even more so when evaluating infectious outcomes such as HAI. One study, by Piatti et al,^22^ used a cross-classified MLM to assess the unit-level risk of *C. difficile* transmission in a Northern Italian hospital.^22^ The researchers found no evidence of unit-level effects, demonstrating homogeneity in risk of infection across units. This research design differs substantially from ours, particularly in that it allowed for community-acquired infections (a community clustering unit). In general, we must also consider that a hospital’s guidelines for infection prevention, patient populations and risk profiles, and baseline levels of HAI transmission, often differ based on internal and external factors that we may not have accounted for. Additionally, our study focused solely on patients within acute care hospitals and may not be relevant to long-term acute care patients, who are known to be at increased risk of *C. difficile* infection.^8^ Thus, we would expect results to vary depending on the study population, time-period of follow-up, and hospital type. However, concerns about correct specification of time-varying risk factors and clusters would remain relevant regardless of contextual factors and underlying study population. We therefore recommend that researchers interested in assessing environmental risk factors for HAIs consider how their patients may be moving throughout or between units and apply methods such as cross-classified MLMs to better account for these changing environments.

In a more similar study population and setting, Arora et al^13^ addressed similar concerns about time-varying risk factors in HAI research in their 2016 paper on healthcare-associated *C. difficile* infection.^13^ As with our results, the authors found that antibiotic exposure was the strongest predictor of *C. difficile* infection, followed by the number of antibiotics prescribed by unit. This study did not quantify the amount of variation in the outcome between units, yet they did include one unit-level covariate (antibiotic exposure). The authors identify the opportunity to consider additional unit-level covariates, such as average unit-level immunity, network structure, or physical structure of the unit. The lack of available information for evaluating unit-level risk factors is one of the limitations of our work. In our analyses, the unit cluster acts as a proxy for unit-level risk factors, yet we are unable to determine the specific characteristics of the hospital units that may be driving the residual heterogeneity in odds of infection between units. In addition to those mentioned by Arora, future research should consider environmental cleanliness, hand hygiene, physical structure of rooms (single vs. multiple occupancy), and adherence to infection prevention protocols as unit-level risk factors.

Another limitation is the timing of our study, since the data were collected during the COVID-19 pandemic. Numerous factors that occurred during the pandemic may have influenced the incidence of *C. difficile,* such as increased hand hygiene, social distancing, and patient isolation, as well as fewer non-COVID-19 admissions and changes in antibiotic prescribing practices.^23^ Due to a change in hospital *C. difficile* testing procedures from only PCR testing to PCR + immunoassay testing at the end of 2019, we chose to use data from this period despite the pandemic to avoid conflicting specifications in case definition. We recognize that these cases may perhaps be sicker than cases who would have been admitted prior to the pandemic, which could be why we are seeing such large associations between antibiotic exposure and *C. difficile* infection. If the data are available, future research should evaluate if differences in associations between patient-level or unit-level risk factors and hospital-associated *C. difficile* infection exist before, during, or after the pandemic.

Finally, the magnitude of change in the effect estimates calculated in the original vs. the lagged sensitivity analyses is larger than hypothesized. Incubation time for *C. difficile* is not well characterized, and there is little research on the appropriateness of using a lag to measure time of infection.^20^ Our results suggest that as time passes the impact of the measured time-varying factors on risk of infection diminishes in this population.

Healthcare-associated *C. difficile* infection is complex, with multiple levels of risk factors influencing a patient’s probability of infection and subsequent disease. Accounting for clustering of patients within physical spaces will produce more accurate estimates of the association between risk factors and *C. difficile* infection, as well as allow for the calculation of the potential impact a patient’s environment may have on their risk of infection. Using hierarchical and cross-classified MLMs, we found that while antibiotic use is the most important risk factor in patients that developed healthcare-associated *C. difficile* infection, environmental risk factors are additionally important and should be considered in research involving hospitalized patients and HAI.

## Supporting information

Webster et al. supplementary material Webster et al. supplementary material

## Supplementary material

The supplementary material for this article can be found at https://doi.org/10.1017/ice.2025.10356
