# Supplementary material for: Estimating the impact of patient-level risk factors and time-varying hospital unit on healthcare-associated Clostridioides difficile infection using cross-classified multilevel models
Source: Infect Control Hosp Epidemiol. 2025 Dec 1;47(2):145–52. doi: 10.1017/ice.2025.10356 (PMC12926340; doi:10.1017/ice.2025.10356)
Supplement: Webster et al. supplementary material [file S0899823X25103565sup001.docx]

**Supplemental Document 1**

**Methods that account for spatial- and time-unit clustering**

To determine the most appropriate approach to our analysis considering our research question, study population, and data availability, we summarized the types of methods used for evaluating data that include a spatial clustering unit, a time clustering unit, or both. While we did not include spatial proximity of units in our analyses, we are considering the physical location of patients as their “spatial” cluster and that visiting a given unit inherently encompasses a spatial dimension of exposure. Within these three cluster constructs, **Table 1** describes the use of traditional generalized linear models (GLM), hierarchical multilevel models (MLM), and non-hierarchical MLM, and includes a description of the data structure, covariates considered, and advantages and disadvantages for using each method, in the context of our research question. The most important consideration for model selection is the overall research objective. For example, the goal of the research may be to evaluate differences in a patient’s risk of the outcome based on patient-level risk factors, environmental risk factors, or both, in which case models that include covariates representing risk factors of interest would be the preferred analytic method. Alternatively, the goal may be to evaluate only patient-level risk factors for the outcome while accounting for non-independence or correlation within clusters, in which case a MLM with patients nested within the cluster and including the patient-level risk factor as a covariate would be the preferred analytic method. Another important consideration is data structure and availability – i.e., what information is accessible, at which level of the data, and how can it be meaningfully represented? This could relate to the types of covariates available (patient-level or unit-level), as well as how they are measured within the dataset (summary measures specific to each patient, or measured daily and varying over time).

In the context of this research, with the goal of evaluating the patient-level risk factors for *C. difficile* infection, taking into consideration clustering at the environmental (unit) level, we chose to use a multilevel approach with patients nested within units. We begin the analysis with a two-level MLM, where patients were nested within the unit where they spent the longest amount of time. This model would allow for a hierarchical structure, by using a summary measure of the time-varying unit cluster visited This model assumes that the unit the patient spent the longest amount of time would likely contribute the most to their probability of exposure and risk of the outcome. This assumption may not be appropriate for all exposures and outcomes, and as such, other more targeted summary measures could be utilized instead. As an alternative method, to incorporate the time-varying unit, we conducted a (non-hierarchical) cross-classified MLM, ^1^ with patient-day as the smallest unit of analysis to account for the time-varying covariates and unit cluster, with patient-day nested simultaneously (but not hierarchically) within patient and unit.

In these analyses, the unit cluster acts as a proxy for the environmental risk factors, and treating it as a cluster as opposed to a covariate will allow the model to adjust for these factors, for the correlation between patients within the same units, as well as to calculate the amount of variation in the outcome observed between units. Additionally, as the current dataset includes many clusters (17 units) and our outcome of interest is relatively rare, including unit as a covariate would violate the positivity assumption as some units did not include any patients that experienced the outcome. ^2^

**Multilevel Model Power Calculation**

Power calculations were conducted prior to data collection to determine the range in values of power that will be achievable given different sample sizes for a cross-classified multilevel model. We assumed a fixed two-sided alpha of 0.05, a desired β (probability of a type II error) of 0.20, an estimated effect size of 0.8, an estimated patient-level variance of 5 and an estimated unit-level variance of 0.4. We ran 1,000 simulations using the Zero/One method to estimate the power for a range of sample size combinations. ^3^ The results of this power calculation are provided in the table below. These calculations were conducted using MLPowSim Software Package (University of Bristol).

| **Table.** Power estimates for a cross-classified multilevel model. | | | |
| --- | --- | --- | --- |
| **Average # of transfers per patient** | **Average # of patients per unit** | **Average # of units** | **Power (95% Confidence Interval)** |
| 1 | 100 | 2 | 0.397 (0.37,0.43) |
| 1 | 100 | 6 | 0.637 (0.61, 0.67) |
| 1 | 100 | 10 | 0.71 (0.68, 0.74) |
| 1 | 300 | 2 | 0.618 (0.59, 0.65 |
| 1 | 300 | 6 | 0.798 (0.77, 0.82) |
| 1 | 300 | 10 | 0.905 (0.89, 0.92) |
| 1 | 500 | 2 | 0.691 (0.66, 0.72) |
| 1 | 500 | 6 | 0.838 (0.82, 0.86) |
| 1 | 500 | 10 | 0.949 (0.94, 0.96) |
| 3 | 100 | 2 | 0.561 (0.53, 0.59) |
| 3 | 100 | 6 | 0.637 (0.61, 0.67) |
| 3 | 100 | 10 | 0.762 (0.74, 0.79) |
| 3 | 300 | 2 | 0.637 (0.61, 0.67) |
| 3 | 300 | 6 | 0.793 (0.77, 0.82) |
| 3 | 300 | 10 | 0.907 (0.89, 0.93) |
| 3 | 500 | 2 | 0.692 (0.66, 0.72) |
| 3 | 500 | 6 | 0.838 (0.82, 0.86) |
| 3 | 500 | 10 | 0.945 (0.93, 0.96) |

Because the average number of transfers per patient was 2.1, the average number of patients per unit was 165, and the total number of units 16, we are confident in the power of our multilevel model results.

**Multilevel model equations**

For Model 1 (hierarchical MLM), we first specified an unconditional (empty) model, denoted as:

*Equation 1*  $Y_{ij} \sim Binomial(1, \pi_{ij})$

$$logit(\pi_{ij})=\gamma_{00}+U_{0j}$$

Where $\pi_{ij}$ is the probability that patient $i$ in longest unit $j$ is diagnosed with *C. difficile* infection, $\gamma_{00}$ is the average log odds of infection in longest unit $j$, $U_{0j}$ is the deviation for each longest unit from the log odds of infection in the average longest unit, and $U_{0j}$ is assumed to be normally distributed with a mean of 0 and a variance of $\tau_{00}$. This model was then extended to include patient-level covariates. The conditional Model 1 is denoted as:

*Equation 2* $Y_{ij} \sim Binomial(1, \pi_{ij})$

$$logit\left( \pi_{ij} \right)=\gamma_{00}+\gamma_{10}{Antibiotics}_{ij}+\gamma_{20}{Medications}_{ij}+\gamma_{30}{Procedures}_{ij}+\gamma_{40}{Age}_{ij}+\gamma_{50}{Insurance}_{ij}+\gamma_{60}{AHRQ}_{ij}+\gamma_{70}{LOS}_{ij}+\gamma_{80}{Transfers}_{ij}+U_{0j}$$

Where ${Antibiotics}_{ij}$, ${Medications}_{ij}$, ${Procedures}_{ij}$, ${Age}_{ij}$, ${Insurance}_{ij}$, ${AHRQ}_{ij}$, ${LOS}_{ij}$, and ${Transfers}_{ij}$ are the patient-level covariates for the $ith$ patient in the $jth$ unit. $\gamma_{10}$-$\gamma_{80}$ are the unit-level intercepts (fixed effects) of the patient-level covariates. This model accounts for correlation between patients that spent the longest amount of time in the same units, but ignores any potential correlation between patients within units where they spent less time.

For Model 2, the unconditional model was denoted as:

*Equation 3* $Y_{i(jk)} \sim Binomial(1, \pi_{i(jk)})$

$$logit(\pi_{i(jk)})=\gamma_{00}+U_{00j}+U_{00k}$$

Where $\pi_{i(jk)}$ is the probability of *C. difficile* infection on day $i$ for patient $j$ in unit $k$. $\gamma_{00}$ is the average log odds of infection over all patient-days. $U_{00j}$ is the random effect of patient $j$ averaged across all units. $U_{00j}$ is assumed to be normally distributed with a mean of 0 and a variance of $\tau_{j00}$. $U_{00k}$ is the random effect of unit $k$ averaged across all patients. $U_{00k}$ is assumed to be normally distributed with a mean of 0 and a variance of $\tau_{k00}$. This model allowed us to determine the extent of unit-level differences in *C. difficile* infection.

Model 2 was also extended to include patient-day-level (time-varying) and patient-level covariates. Covariates were characteristics identified a priori as potential risk factors for *C. difficile* infection, based on prior research demonstrating a clinical and epidemiological significance. The conditional model is denoted as:

*Equation 4*  $Y_{i(jk)} \sim Binomial(1, \pi_{i(jk)})$

$logit\left( \pi_{i\left( jk \right)} \right)=\gamma_{00}+\gamma_{10}{Antibiotics}_{i\left( jk \right)}+\gamma_{20}{Medications}_{i\left( jk \right)}+\gamma_{30}{Procedures}_{i\left( jk \right)}+\gamma_{40}{LOS}_{i\left( jk \right)}+$ $\pi_{01}{Age}_{jk}+\pi_{02}{Insurance}_{jk}+\pi_{03}{AHRQ}_{jk}+\pi_{04}{Transfers}_{jk}$

$$+U_{00j}+U_{00k}$$

Where ${Antibiotics}_{i(jk)}$, ${Medications}_{i(jk)}$, ${Procedures}_{i(jk)}$, and ${LOS}_{i(jk)}$ are patient-day-level (time-varying) covariates for the $ith$ patient-day in the $jth$ patient in the $kth$ unit; ${Age}_{jk}$, ${Insurance}_{jk}$, ${AHRQ}_{jk}$, and ${Transfers}_{jk}$ are the patient-level (time-invariant) covariates for the $jth$ patient in the $kth$ unit. $\gamma_{10}$-$\gamma_{40}$ are the unit-level intercepts (fixed effects) of the patient-day covariates. $\pi_{01}$-$\pi_{04}$ are the unit-level intercepts (fixed effects) of the patient covariates. This model allowed us to determine how much unit-level differences in *C. difficile* infection could be explained by patient-level risk factors.

**References**

1. Fielding A, Goldstein H. *Cross-classified and multiple membership structures in multilevel models: An introduction and review*. Department for Education and Skills London; 2006.

2. Petersen ML, Porter KE, Gruber S, Wang Y, van der Laan MJ. Diagnosing and responding to violations in the positivity assumption. *Stat Methods Med Res*. Feb 2012;21(1):31-54. doi:10.1177/0962280210386207

3. Browne, W.J., M.G. Lahi, and R.M. Parker, A guide to sample size calculations for random effect models via simulation and the MLPowSim software package. Bristol, United Kingdom: University of Bristol, 2009.
